# Solving difficult problems creatively: a role for energy optimised deterministic/stochastic hybrid computing

**DOI:** 10.3389/fncom.2015.00124

**Published:** 2015-10-12

**Authors:** Tim N. Palmer, Michael O’Shea

**Affiliations:** ^1^Department of Physics, University of OxfordOxford, UK; ^2^Centre for Computational Neuroscience and Robotics, University of SussexBrighton, UK

**Keywords:** deterministic and stochastic simulations, slender axons, creative thinking, energy-optimised computing, inexact computing

## Abstract

How is the brain configured for creativity? What is the computational substrate for ‘eureka’ moments of insight? Here we argue that creative thinking arises ultimately from a synergy between low-energy stochastic and energy-intensive deterministic processing, and is a by-product of a nervous system whose signal-processing capability per unit of available energy has become highly energy optimised. We suggest that the stochastic component has its origin in thermal (ultimately quantum decoherent) noise affecting the activity of neurons. Without this component, deterministic computational models of the brain are incomplete.

Problems that are computationally complex can be routinely solved by the nervous system with remarkably little expenditure of energy. Consider for example the Travelling Salesman task. Computationally this is notoriously difficult to solve because the number of candidate “shortest routes” increases exponentially with the number of destinations and purely deterministic algorithms can take an unacceptably long time to reach solution. And yet bumblebees foraging on arrays of flowers optimise their flight distances and rearrange their flower visitation sequences dynamically as new sources of food are presented (Lihoreau et al., 2010). It is well known that heuristic (i.e., simplified) algorithms that combine stochasticity and determinism can frequently outperform purely deterministic algorithms for solving such combinatorial problems (Gomes et al., 1998; Hoos and Stützle, 2005). Here we propose that signal processing in energy-constrained nervous systems have evolved by combining low-energy stochastic and energy-intensive deterministic computation in a highly energy-optimised way. As a by-product, we speculate that the evolution of such hybrid deterministic/stochastic computing capability in biological systems provides the fundamental physical basis for creative problem solving in the human brain.

Our proposal for an energy-optimised hybrid stochastic/deterministic computational model for the operation of the brain is inspired by the development of a new type of energy-optimised computer which operates in both probabilistic and conventional bit-reproducible mode (Palem, 2003, 2014; Chakrapani et al., 2006). One motivation for this development is that, as the density of microprocessors in computers increases and individual transistors approach atomic scale, the power needed to ensure microprocessors operate deterministically is becoming unsustainable. By relaxing the constraints which ensure transistors are immune to thermal noise, it is possible to design microprocessors that operate probabilistically rather than bit-reproducibly with a considerable reduction in energy consumption. There are many problems in computational science—typically those governed by chaotic dynamics—where solutions can only be determined in some probabilistic sense and therefore do not need strictly deterministic computing capabilities. For a given energy resource and problem class, it is possible to design systems for which the ratio of deterministic to probabilistic computing elements is optimised. Combining energy-efficient probabilistic processors with energy-intensive deterministic processors in such an optimised way can provide new and efficient hybrid tools for solving complex (and otherwise intractable) computational problems (Palmer, 2012), including the type of combinatorial problem discussed above, for a given energy resource.

A particular example of a problem class which can benefit from such hybrid computation, with potential links to understanding the nature of human creativity, is that of finding the global minimum of some objective function. Purely deterministic heuristic algorithms risk converging to some local minimum. This risk is reduced in partially stochastic schemes (such as simulated annealing) which can jump randomly from one local basin to another. More generally, adding an element of stochasticity to an otherwise deterministic heuristic can help prevent the occurrence of problem instances where the time to solution becomes unacceptably long, thus improving the overall performance of the heuristic (Gomes et al., 1998).

In the brain, information is transmitted in the temporal pattern of action potentials (spikes or nerve impulses). But the brain is susceptible to a variety of sources of noise which affect the reliability of the information contained in spike trains (White et al., 2000; Faisal et al., 2008; Rolls and Deco, 2010). An important example of noise operating at the molecular level can be seen in the behaviour of voltage sensitive ion channels or “protein transistors”, which amplify electrical signals in neurons and are collectively responsible for generating the propagating action potential. These ion channels open and close deterministically in response to voltage fluctuations, but they are also subject to thermal noise (Chow and White, 2000; McDonnell and Ward, 2011) that results in their random opening and closure (ion channel noise). In neurons with axon diameters greater than 1μm however, channel noise does not significantly corrupt the information content of spike trains (Faisal and Laughlin, 2007; Sengupta et al., 2013) because the relatively minute electrical signals generated by individual ion channels will not reach the threshold for spike generation. Larger neurons therefore are reliably deterministic. Larger neurons also transmit information more rapidly due to their lower resistance to axial current flow. Speed and reliability however are costly because large neurons are relatively energy inefficient. This is because larger neurons necessarily have lowered input resistance and therefore require more ionic current to trigger a nerve impulse. Following a bout of impulses, critical ionic concentrations across the neuronal membrane must be restored by energy-consuming ionic pumps. The larger the axon the more work will need to be done to recharge the ionic batteries on which impulse generation depends. In neurons with the most slender dimensions, speed of information transmission is sacrificed for the benefit of increased energy efficiency. Smaller neurons are more efficient because their high input resistance allows relatively small trans-membrane ionic currents to generate sufficient trans-membrane voltage to trigger a nerve impulse. Indeed if the local input resistance is high enough, current flowing through a single ion channel may be sufficient to generate an impulse. As single ion channels are susceptible to thermal noise, the advantages of smallness (efficiency and density of computing elements for example) may (as in transistors with reduced guardband voltage) be accompanied by a greater probability of stochastic corruption of the temporal pattern of impulse trains. Overall then, thermal noise affecting voltage sensitive ion channels is likely to decrease the reliability of impulse timing especially in fine axons when energy supply is restricted.

The miniaturisation of the brain’s wiring permits a significantly larger number and density of signal-processing elements (i.e., neurons) than would otherwise be possible (Faisal et al., 2005). Based on the developments in computer science outlined above, we suggest this may be accompanied by substantial beneficial consequences for the brain’s computational performance, over a more traditional deterministic perspective. These include increased computational performance per unit of energy expended, a smaller likelihood of algorithmic “hanging” when making decisions, and as discussed below, the potential for what is generally referred to as “creativity” in problem solving. In developing this argument we suppose that the brain’s evolution has proceeded unconstrained by assumptions about how it ought to work—for example to operate as fault free as possible. So, if there were fitness benefits to be gained by expending available energy to power hybrid probabilistic and deterministic signal-processing hardware in the brain, natural selection would have exploited them. We propose that the advantages of combining stochastic and deterministic computing can be exploited by any species possessing a sufficiently large and complex nervous system. So for example we would suggest that problem solving in the bee, with a brain containing about one million highly miniaturised neurons, is facilitated by noise arising in its small energy efficient neurons. This is not to claim however that in solving the travelling salesman problem, the bee is behaving creatively. While being difficult to define with precision, the term “creativity” implies conscious engagement with problem solving tasks and this property is believed to have appeared late in the evolutionary line leading to modern humans. So, we would propose, only when the evolving brain reached the size and complexity of the primate brain—probably in the common ancestor of man and chimps, some 7–10 million years ago—did conscious creativity emerge from the synergy between stochastic and deterministic energy-optimised hybrid neural computing. But, we suggest, hybrid neural computing evolved much earlier because it provides animals (conscious or not) with the selective advantage of energy efficient solutions to combinatorial problems encountered in an unforgiving environment (such as is occupied by the bee, for example). To make our position clear, we believe the hybrid model we propose serves as a general substrate for conscious creativity in human as well as for adaptive problem solving in other species. From this discussion, it might seem reasonable that neuron miniaturisation (Niven and Farris, 2012) and the accompanying obvious benefit of higher packing density of computing elements, is limited by the trade-off between energy efficiency and the need to preserve information coding at an acceptable rate and reliability. There is however an alternative interpretation of miniaturisation, namely that it allows the advantages of the relative un-reliability of information transmission by small neurons to be exploited. Here we suggest that neurons with axon diameters around 0.1 μm introduce this useful, energy efficient, stochastic component to neural computations.

We are not the first to propose a constructive role for stochasticity in simulating the brain (see e.g., White et al., 2000). Indeed, in his famous paper on “Computing Machinery and Intelligence”, whose first Section defines the famous “Imitation Game”, Turing (1950) notes (p. 438): “It is probably wise to include a random element in a learning machine” and discusses the example of finding a number (say between 50 and 200) equal to the square of the sum of its digits. A systematic deterministic approach has the disadvantage that there will be enormous blocks of numbers without any solution. Investigation of these blocks in a deterministic scheme is an instance of a situation, discussed above, where such schemes can take an unacceptably long time to reach a solution.

More recently the phenomenon of ‘Stochastic Resonance’ or SR has been cited as a mechanism by which noise in sensory systems enhances sensitivity (Wiesenfeld and Moss, 1995). In SR, random noise associated with a marginally sub-threshold periodic signal increases the probability that repetitive bursts of spikes at the signal period will be generated. However, SR is a mechanism for making the electrical activity of neurons more reliably deterministic—not less so. This example supports the conventional dichotomy in which noise is either beneficial for deterministic operation (SR) or is a nuisance (channel noise in very small neurons). Here, however, we propose something more in keeping with Turing’s proposal: that the inherently probabilistic character of signal corruption by channel noise, a consequence of evolution towards highly energy efficient signal-processing nervous systems, actually contributes positively to brain function.

Could a model which combines stochasticity and determinism in some synergistic hybrid operation which is optimised for energy efficiency, be relevant to the human brain? Certainly as the brain is an energetically “expensive” organ (Laughlin et al., 1998; Attwell and Laughlin, 2001), it is reasonable to believe it will have evolved to favour mechanisms that consume energy efficiently. Motivated in part by the fast/slow thinking dichotomy of Kahneman (2012), consider for simplicity two cognitive modes, referred to as Mode 1 and Mode 2. Mode 1 is an economical, relatively low-energy mode which maintains function across small, energy-efficient but slower neurons which are susceptible to thermal noise. In Mode 2, available energy is focussed on a less energy-efficient subset of neurons ensuring that they operate reliably, quickly and hence deterministically. Although signal propagation in Mode 2 is relatively fast, the time taken to process data deterministically and hence precisely in Mode 2 will be relatively slow.

Continually switching between Mode 1 and 2 is reminiscent of the way a stochastic heuristic algorithm (such as discussed above) tackles the problem of finding the global minimum of an objective function: stochasticity allows some jumping between local “potential wells”, and minimises the chance that the heuristic does not “get stuck” near some local minimum. By analogy, in trying to find the solution to a problem which requires creative thinking (for example, proving that the square root of 2 is either rational or irrational) the human brain might start in Mode 1, randomly selecting a candidate line of enquiry (e.g., properties of the geometry of circles—representing a local region in some abstract space of mathematical concepts), and exploring the logical developments in Mode 2. Influenced by a first unsuccessful attempt to find the solution (c.f. failing to find the global minimum) a second candidate line of enquiry can again be randomly chosen in Mode 1 (e.g., properties of even numbers) and the required solution found through further analysis in Mode 2.

Consistent with this, it is a familiar experience that taking a break in concentration from some difficult problem, i.e., switching from Mode 2 to Mode 1, can provide unexpected new angles on a problem at hand, ultimately leading to its solution. The mathematical physicist Roger Penrose (Penrose, 1994) has documented a number of classical ‘eureka moments’ when a scientist (himself included) was engaged in otherwise mundane activity, such as crossing the road or stepping onto a bus. If, in some non-deterministic way, a potential insight occurs when the brain is operating in Mode 1, randomly switching between local basins in some conceptual state space, it is often straightforward to check using Mode 2 that this insight does indeed solve the problem.

We conclude with some comments about links to artificial intelligence. Firstly, from a theoretical point of view, our hybrid probabilistic/deterministic computing system provides a novel way to understand the implications of Gödel’s Theorem. Gödel’s theorem is essentially a statement about the incompleteness of algorithmic reasoning: no matter how complex a formal logical system might be, there are always logically sound propositions that cannot be proven by deductive reasoning using the rules of the system. By definition, Gödel’s Theorem would be meaningless to an artificial intelligence that operates by deterministic computational (and hence algorithmic) rules. That Gödel’s theorem is not meaningless to us humans is suggestive of the fact that we do not operate entirely by such computational rules. Penrose argues that because of Gödel’s theorem, the human brain cannot be emulated by a conventional digital computer, no matter how big (Penrose, 1994). He argues from this that coherent quantum entanglement effects must therefore be operating in the brain. However, there is little support for Penrose’s thesis within the neuroscience community (Baars and Edelman, 2012); not least it is believed that quantum entanglement can play no significant role in the action of the brain because decoherence timescales in the warm noisy environment of the brain would prevent isolated entanglements from lasting long enough to be relevant for dynamical neural timescales.

However, the Penrose argument is readily accounted for by our hybrid probabilistic/deterministic proposal for the operation of the brain, precisely because the ultimate source of neuronal noise at the molecular level is quantum decoherence. The term “decoherence” describes the random nature by which a quantum system reveals its properties when interacting with its environment. Here the word “random” encompasses the notion that, under repeated preparation and measurement, the fluctuations in the measured properties of a quantum system do not appear to be representable with any finite algorithm (Calude et al., 2010). Because it is, on occasion, susceptible to such random quantum mechanical noise, the cognitive action of the brain cannot by definition be represented by finite algorithm—consistent with human understanding of Gödel’s Theorem.

From the perspective of the hybrid probabilistic/deterministic proposal, it is reasonable to suppose that the more random and more constant the source of noise, and the less energy needed to access it, the more effective it will be for complex problem solving. In computational science, stochastic search algorithms are often driven by integrating low-order chaotic systems over extended periods of time (Hoos and Stützle, 2005). Such systems are neither genuinely random, nor entirely cost free to integrate, and in any case, there is no evidence that low-order chaos features in the operation of the brain. On the other hand, over the years, a large body of theoretical literature has demonstrated that the dynamics of neural networks can operate as a high-dimensional chaotic system (van Vreeswijk and Sompolinksy, 1996; Monteforte and Wolf, 2010). Could output from such a system be a viable alternative to quantum decoherence in providing a reliable source of randomness for problem solving in the brain? Some factors argue against this, though with the current state of knowledge it is not possible to be definitive about this. Firstly, in a nonlinear dynamical system, local Lyapunov exponents will vary with position on the attractor (Palmer, 1993), and consequent noise output from a high-dimensional deterministic chaotic system cannot in general be expected to be constant in time (the intermittency of fluid turbulence being a specific example). Secondly, it is not immediately clear how high-dimensional deterministic chaos would account for deterministic signal propagation along thick axons and more stochastic propagation along slender axons. By contrast, quantum decoherent noise is as random as it is possible to be (Calude et al., 2010), is ubiquitous in both space and time and, by virtue of the fact that it operates primarily on molecular (and smaller) scales, affects slender axons more than thick ones. And crucially, since susceptibility to quantum decoherent noise depends inversely on the energy expended in propagating signals along axons, one is actually saving energy by making signal propagation susceptible to quantum decoherent noise.

These remarks are relevant for attempts to emulate the brain on next-generation exascale computers. Notwithstanding the fact that such computers may require in excess of 50MW to operate (Kogge, 2008) and hence will need several orders of magnitude more energy than the brain itself needs, we suggest that if quantum decoherent noise is an essential element in the operation of the brain, the brain will not be fully emulated on an energy-constrained deterministic machine. Indeed, we propose that simulations of the brain should be conducted on a new class of energy-optimised imprecise supercomputer (Palmer, 2015) which actually mimics the hybrid deterministic/stochastic structure on which, we have suggested, the brain itself is based.

## Conflict of Interest Statement

The authors declare that the research was conducted in the absence of any commercial or financial relationships that could be construed as a potential conflict of interest.
